# Cognitive Modeling of Automation Adaptation in a Time Critical Task

**DOI:** 10.3389/fpsyg.2020.02149

**Published:** 2020-10-02

**Authors:** Junya Morita, Kazuhisa Miwa, Akihiro Maehigashi, Hitoshi Terai, Kazuaki Kojima, Frank E. Ritter

**Affiliations:** ^1^Department of Behavior Informatics, Faculty of Informatics, Shizuoka University, Hamamatsu, Japan; ^2^Department of Cognitive and Psychological Sciences, Graduate School of Informatics, Nagoya University, Nagoya, Japan; ^3^Center for Research and Development in Admissions, Shizuoka University, Shizuoka, Japan; ^4^Department of Information and Computer Sciences, Faculty of Humanity-Oriented Science and Engineering, Kinki University, Fukuoka, Japan; ^5^Learning Technology Laboratory, Teikyo University, Tochigi, Japan; ^6^College of Information Sciences and Technology, Pennsylvania State University, University Park, PA, United States

**Keywords:** automated operation, reinforcement learning, ACT-R, Semi-Markov Decision Process, trust calibration

## Abstract

This paper presents a cognitive model that simulates an adaptation process to automation in a time-critical task. The paper uses a simple tracking task (which represents vehicle operation) to reveal how the reliance on automation changes as the success probabilities of the automatic and manual mode vary. The model was developed by using a cognitive architecture, ACT-R (Adaptive Control of Thought-Rational). We also introduce two methods of reinforcement learning: the summation of rewards over time and a gating mechanism. The model performs this task through productions that manage perception and motor control. The utility values of these productions are updated based on rewards in every perception-action cycle. A run of this model simulated the overall trends of the behavioral data such as the performance (tracking accuracy), the auto use ratio, and the number of switches between the two modes, suggesting some validity of the assumptions made in our model. This work shows how combining different paradigms of cognitive modeling can lead to practical representations and solutions to automation and trust in automation.

## 1. Introduction

Automation technology, which can partially substitute for human cognitive functions, has made remarkably progress recently. Although the application area of such technology is diverse, one of the recent prominent areas is the automatic operation of vehicles. The operation of ships and aircraft has commonly been automated in our society. For cars, automation of some functions such as speed control (i.e., adaptive cruise control) and braking (anti-lock) have also been used for a long time. In recent years, automatic control of steering has been actively developed due to the rapid progress of sensing and machine learning technologies. However, there are still barriers to the full application of automatic driving (self-driving cars). For a while, it has been assumed that automatic control will be used with driver's monitoring to intervene immediately at any time if the automatic control fails to respond properly (National Highway Traffic Safety Administration, 2016).

When new technologies, not limited to automatic control of vehicles, are introduced, misuse (overreliance) and disuse (underutilization) of the technologies has often become a problem. For sustainable industrial development in our society (United Nations Industrial Development Organization, 2015; Fukuyama, 2018), it is important to understand how humans adapt to new technologies. Disuse of new technology results in less innovation, while misuse of new technology can cause serious accidents. In the field of human factors, such problems have been repeatedly discussed (e.g., Bainbridge, 1983; Parasuraman and Riley, 1997).

However, previous studies in the field have not fully considered time factors involved in the adaptation process to new technologies. Contrary to some other tasks studied in human factors, vehicle operation is a dynamic continuous process in which the cycle of perception, judgment, and action sequentially repeats. Automated vehicle systems partially substitute for such human operation. In a case where an operator can use automatic operation, s/he repeats the cycle of perception and judgment while observing that an automation system executes the overall cycle. When the operator notices that the auto control has problems, s/he needs to immediately turn off the automation to return to manual control.

We suggest that the above adaptation mechanism to automation technology can be partially explained by reinforcement learning, which updates selection probabilities of actions with rewards from the environment (Sutton and Barto, 1998). In a broader context, this paradigm has already been used to model the interaction between human and automation systems. As will be described in section 2, Gao and Lee (2006) proposed a computational model called Extended Decision Field Theory that simulates how operators adapt to a system that automates plant operation. In their model, a selection probability of using the automation system is dynamically changed through iterated environmental feedback.

However, it is not simple to apply the paradigm of reinforcement learning to a task that involves time-critical decision making. Generally, reinforcement learning has been applied to discrete tasks through Markov Decision Processes (MDP)—like bandit tasks (Sutton and Barto, 1998). Contrary to typically applied fields of reinforcement learning, vehicle operation does not directly fit into a MDP representation. Rather, it can be expressed as a SMDP (Semi-Markov Decision Process). SMDP introduces the concept of a time delay between action selection and state transition, and rewards that can be delivered at different points in time (Duff and Bradtke, 1996; Asada et al., 1999; Sutton et al., 1999; Georgeon and Ritter, 2012; Rasmussen and Eliasmith, 2013).

In this paper, we present a simple task that has some characteristics of continuous vehicle operation with automation and construct a model to reveal what type of mechanisms can simulate human adaptation to automation in a time-critical task like automatic vehicle operation. We specifically try to answer this question integrating a traditional reinforcement learning algorithm with a cognitive architecture. Using a cognitive architecture, we will explore this question using appropriate time constraints on behavior.

In the next section, we review research associated with the present study, showing a framework to simulate human behavior in real-time, and research on adaptation to automated systems. After that, we present the task, initial human data, the model, and the simulation results, describing a mechanism that can simulate a human adaptation process to automatic vehicle operation and show how it predicts and explains human performance on the task. Finally, we summarize the results obtained in this study and their implications.

## 2. Related Studies

This section presents previous work relating to the method and the tasks of the previous studies that define the theories and models that we use.

### 2.1. Research About Cognitive Architecture

Many reinforcement learning studies have been conducted so far. Currently, this approach is being used as a framework for end-to-end simulation in tasks such as games and maze search (Silver et al., 2016; Banino et al., 2018). However, these simulations are still unclear in terms of their correspondence with human behavior.

In contrast, cognitive architectures can be regarded as a framework to simulate human behavior with detailed time predictions. Usually, cognitive architectures include modules responsible for perception and action to model the overall behavior in a task. Among several cognitive architectures that have been proposed so far, the present study focuses on ACT-R (Adaptive Control of Thought-Rational; Anderson, 2007; Ritter et al., 2019 for a recent review) because this architecture has a large community and the mechanisms are well-tested. The present study aims to extend such mechanisms to the time-critical domain. Other architectures such as Soar (Laird, 2012) and EPIC (Kieras and Meyer, 1997) could probably be used to simulate this phenomenon as well. Kotseruba and Tsotsos (2020) review numerous other architectures.

So far, ACT-R has been used to model many tasks in the fields of human factors including driving (Salvucci, 2006), teleoperation (Ritter et al., 2007), and air-traffic control tasks (Byrne and Kirlik, 2005; Taatgen, 2005). The rule utility learning mechanism of this architecture also has been applied to simple decision making (Fu and Anderson, 2006) and strategy selection tasks (e.g., Lovett and Anderson, 1996). However, few studies are applying this architecture to the problem of automation use especially in the time-critical field. In the present study, we describe behavioral constraints in a model of automation use with the ACT-R cognitive architecture.

### 2.2. Models of Automation Use

**Figure 1:**
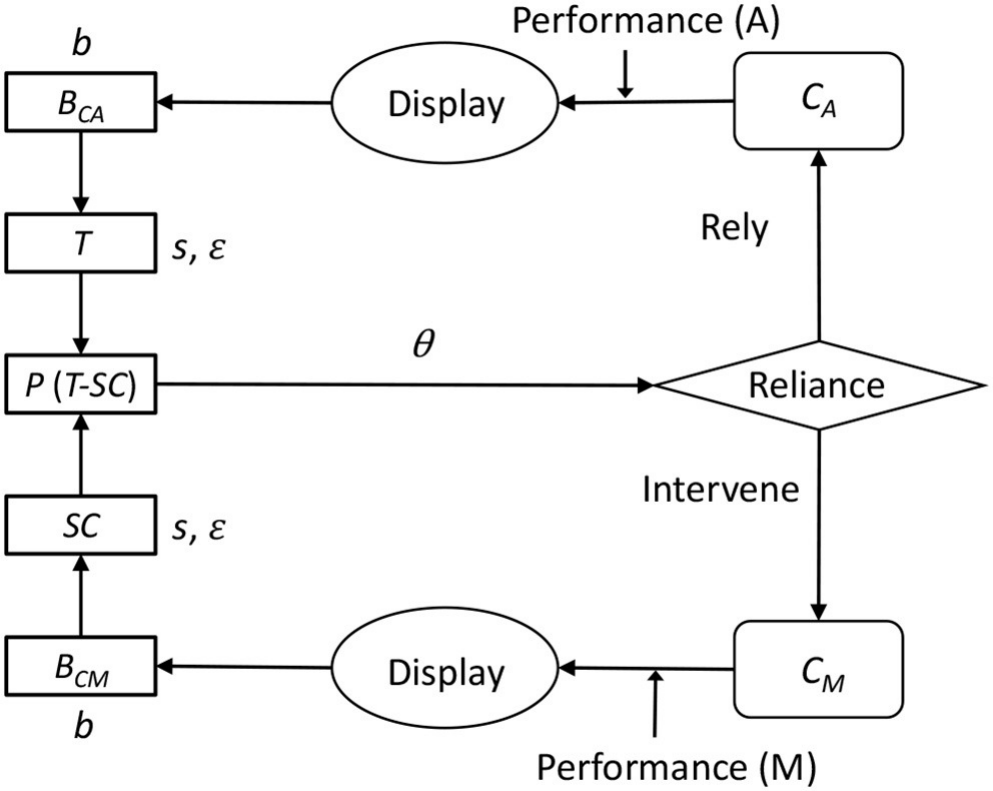
The process of Extended Decision Field Theory (Gao and Lee, 2006), modified and reproduced from the original figure.

In the field of human factors, it has been discussed that automation systems cannot replace human cognitive tasks completely. Bainbridge (1983) claimed that even highly automated systems need human operators to monitor system performance and to make decisions of system use. Some researchers have also pointed out that human decision-making of system use is not optimal. Parasuraman and Riley (1997) noted that there are two types of maladaptive uses of automation: misuse (the overreliance of automation), and disuse (the underutilization of automation). Some studies indicated that human users have a bias to use automation (misuse, Singh et al., 1997; Skita et al., 2000; Bahner et al., 2008). On the other hand, other research has indicated that human users have a bias toward manual control and away from the use of automation systems (disuse, Dzindolet et al., 2002, 2003; Beck et al., 2009).

de Vries et al. (2003) experimentally indicated that the reliance of automation use is influenced by both the capability of auto controls (*Ca*) and capability of manual controls (*Cm*), which cannot be directly observed by operators, but estimated from the displayed performance of each mode. Extended Decision Field Theory (EDFT theory), proposed by Gao and Lee (2006), explains how *Ca* and *Cm* are estimated by people and have an effect on the reliance on automation. This theory extends psychological decision-making theory (Busemeyer and Townsend, 1993), including the effect of previous decisions in the context of multiple sequential decision processes. Figure 1 shows the basic process of the EDFT theory. The theory is given *Ca* and *Cm* values, and constructs beliefs of *Ca* and *Cm* (*Bca*, *Bcm*) based on partially displayed *Ca* and *Cm* values. From the belief values, the theory constructs trust (*T*) and self-confidence (*SC*). The preference for automation (*P*) is determined by subtracting *T* from *SC*. If *P* exceeds an upper threshold (θ), then the theory turns the current control mode to auto. If *P* falls below a lower threshold (−θ), then the theory turns the current control mode to manual. In every cycle, values of *Bca*, *Bcm*, *T*, and *SC* are updated by difference equations. Through this computation, *T* and *SC* dynamically change to approach the actual *Ca* and *Cm*.

The EDFT theory is an abstract mathematical representation that simply explains the reliance on automation in dynamic situations. The strength of this theory is its generality. It can apply to a wide range of situations involving automation. This theory, however, does not have any knowledge about tasks. It cannot interact with a task environment, and it provides no prediction of human performance. Therefore, the present study tries to implement basic assumptions of the EDFT theory in the ACT-R architecture to make detailed predictions of human behavior on a specific task.

## 3. The Task

**Figure 2:**
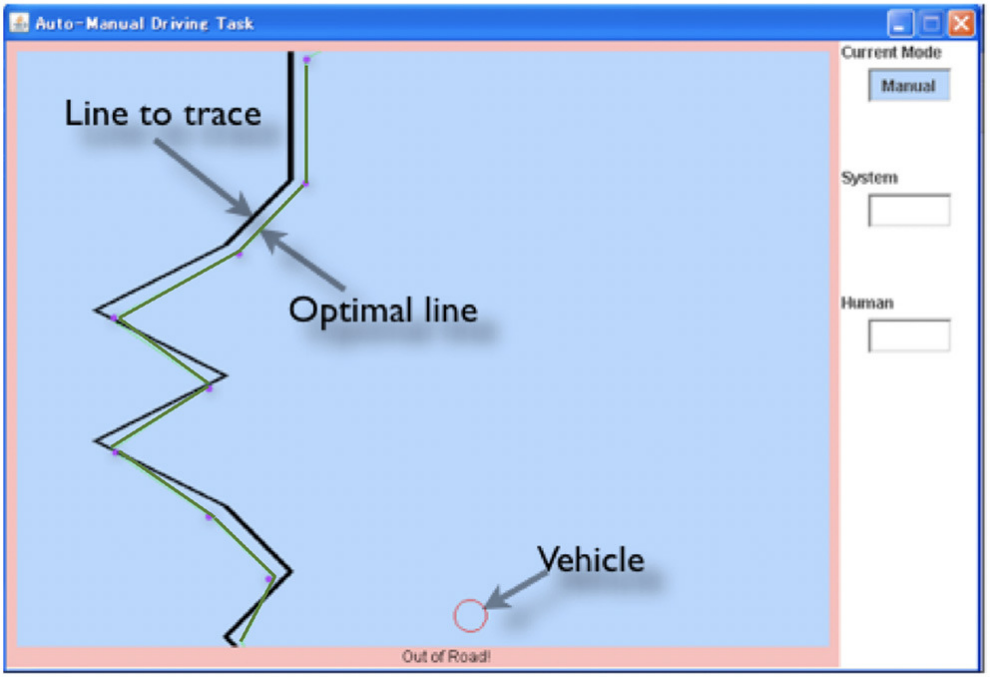
The Line-following task environment.

The present study simulates the behavior of participants in a behavioral experiment conducted by Maeghigashi et al. (2013). The task used in the study was a simple tracking task, called the “line-following task.” Figure 2 shows the task environment. In this task, the operators are required to control the horizontal position of the vehicle (red circle) to follow the black line that scrolls down at 24 pixels per second. The screen is updated every 40 ms. If the vehicle does not overlap the line, a warning is presented outside of the window. The line is drawn by randomly combining 48 pixels high line patterns of varied angles (30, 45, 90, 135, and 150 degrees, equally chosen).

The vehicle is controlled by commands of “left,” “straight,” or “right.” If the vehicle receives a left command, the vehicle moves 1 pixel left from the original position. The command is sampled at 48 Hz. If a key-press event is detected, a flag of sending commands is set to on. This flag is off when a key-release event is detected^1^. If an operator's finger is put on the right arrow key, the vehicle keeps receiving a right command every 20 ms until the key is released. Therefore, maximally, the vehicle can move 2 pixels per one-pixel scroll of the line.

An operator can choose manual or auto controls to send commands. In the manual control, operators use left and right arrow keys to send commands, often several in a row, simulating turning a steering wheel. In the auto control, operators monitor that the auto control moves the vehicle. The auto control tries to follow an optimal line presented as the green line in Figure 2. An optimal line is the shortest line to pass “goals” located on each corner shown as blue dots. If the center of the vehicle is off the optimal line, the auto control system sends a command to correct the vehicle position. In the experiment, the optimal line and goals are not visible to participants.

In both control modes, commands are not always successfully sent to the vehicle. Failures occur at specified rates; *Ca* and *Cm* specify these rates. If *Ca* or *Cm* is low, the vehicle controlled by the corresponding mode is lagged, and it becomes hard to follow the line. To conduct the task successfully, operators need to select a suitable mode in each situation. The operators freely change between modes by pressing the space-bar. The experiment conducted by Maeghigashi et al. (2013) used the task in 25 conditions where *Ca* and *Cm* levels were manipulated (5 levels of *Ca* ranging from 30 to 70% v.s. 5 levels of *Cm* ranging from 30 to 70%). In each condition, the participants (*n* = 63)^2^ performed the task for 40 s.

**Figure 3:**
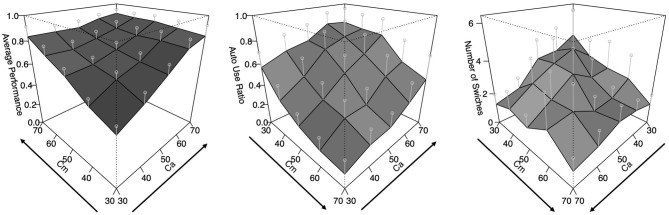
The results of the human experiment. Error bars represent σ/2 (plots are rotated to show data more clearly).

The results of this experiment are summarized as Figure 3. The graph on the left shows the performance of the task, which is the ratio of time that the vehicle was able to follow the line during the task. The middle graph indicates the auto use ratio, which represents how long the auto mode was used during the task. The right graph presents the number of switches, which represents how many switches occurred between the two modes during the task. All graphs have the x-axis and y-axis corresponding to *Ca* and *Cm* levels, respectively, and it can be observed that the values of the z-axis rise from the front to the back. However, the directions of the x and y axes are different in each graph to make the surfaces more visible. For the performance graph, the lowest level of *Ca* and *Cm* is placed in the front. As for the auto use ratio, the highest level of *Cm* and the lowest level of *Ca* are arranged in the front. Concerning the number of switches, both the highest *Cm* and *Ca* levels are placed at the front of the graph.

That is, we can observe that the performance got higher as both *Ca* and *Cm* levels increased. The auto use ratio increased as the level of *Cm* decreased and the level of *Ca* increased. The number of switches increased as the values of *Ca* and *Cm* were lower. The present study tries to construct a cognitive model that reproduces these trends under the same conditions as the human experiment. Although this task is very simple compared to driving in the real-world, the cycle of perception and actions in real-time and the choice to trust automation (or not) is reproduced. We suggest that the task is appropriate to explore adaptation mechanisms to automation in time-critical fields.

## 4. Model

In this section, we will first present the architecture and then the detailed mechanisms of the model will be explained.

### 4.1. Architecture

This study uses ACT-R to construct a model for the line-following task. This architecture integrates several cognitive modules including a visual module, a motor module, and a production module. The visual module is used to take information from an external environment. The motor module manipulates devices like a keyboard or a mouse in an external environment. These modules have buffers to temporarily hold declarative information called a chunk. The production module integrates the other modules by production rules, which consists of a condition/action pair that is used in sequence with other productions to perform a task. Conditions and actions in production rules are specified as patterns in the buffer contents of modules. Further reviews are available (Anderson et al., 2004; Anderson, 2007; Ritter et al., 2019 also see act.psy.cmu.edu).

Importantly, each event executed by ACT-R's modules has a parameter of performance time. For example, ACT-R production rules take 50 ms to apply. Events including visual perception and motor actions, such as eye-movements, mouse-movements and key-presses, also have time parameters. These parameters have been developed and validated by psychological studies (Anderson et al., 2004)^3^. By using these parameters, ACT-R makes real-time simulations possible.

ACT-R also includes sub-symbolic cognitive processes that modulate the probabilities of firing (applying) production rules. When several rules match to the buffer conditions, a rule must be chosen. This is called conflict resolution. The choice is made based on comparisons of the utility values associated with each matching production rule. More specifically, the probability of firing production *i* is described by Equation (1).

(1) $$\begin{array}{r} P\left(i\right)=\frac{e^{U_{i}/\sqrt{2s}}}{\sum_{j}e^{U_{j}/\sqrt{2s}}} \end{array}$$

*s* is a parameter that determines the variance of the noise distribution according to a logistic distribution; *U*_*i*_ is the utility of the production *i* that competes with the number of productions with utility values *U*_*j*_. The learning of *U*_*i*_ is controlled by Equation (2).

(2) $$\begin{array}{r} U_{i}\left(n\right)=U_{i}\left(n-1\right)+\alpha \left[R_{i}\left(n\right)-U_{i}\left(n-1\right)\right] \end{array}$$

α is the learning rate; *R*_*i*_(*n*) is the reward value given to production *i* at time *n*. The learning occurs when a reward is triggered, and all productions that have fired since the last reward are updated. Though the official theory of ACT-R (Anderson, 2007) does not explicitly note it, this learning is the same as the basic reinforcement learning method called Q-learning, which updates the quality of a state-action combination by receiving rewards from the environment (Sutton and Barto, 1998). The relations between the two theories were also discussed by Fu and Anderson (2006).

We considered that the above characteristics (the visual and motor modules to interact with external environments, the real-time simulation, the utility update based on reinforcement learning) are useful for modeling an adaptation process on automatic vehicle operation.

### 4.2. Simulated Task Environment

By using the ACT-R Graphical (user) Interface (AGI) tool that is part of ACT-R 6 (Anderson, 2007; as a technical reference see Bothell, 2007)^4^, we developed a simulated task environment with which the constructed model interacts. The simulated environment is the same as the original environment in the keyboard layout, screen update rates, line scrolling speed, vehicle size, line width, and screen size. The auto control mode is implemented with Common Lisp in the simulated task environment. However, unlike the original environment, visible goal positions are set at each corner to allow the model to perceive the path.

### 4.3. Basic Cycle of the Model

**Figure 4:**
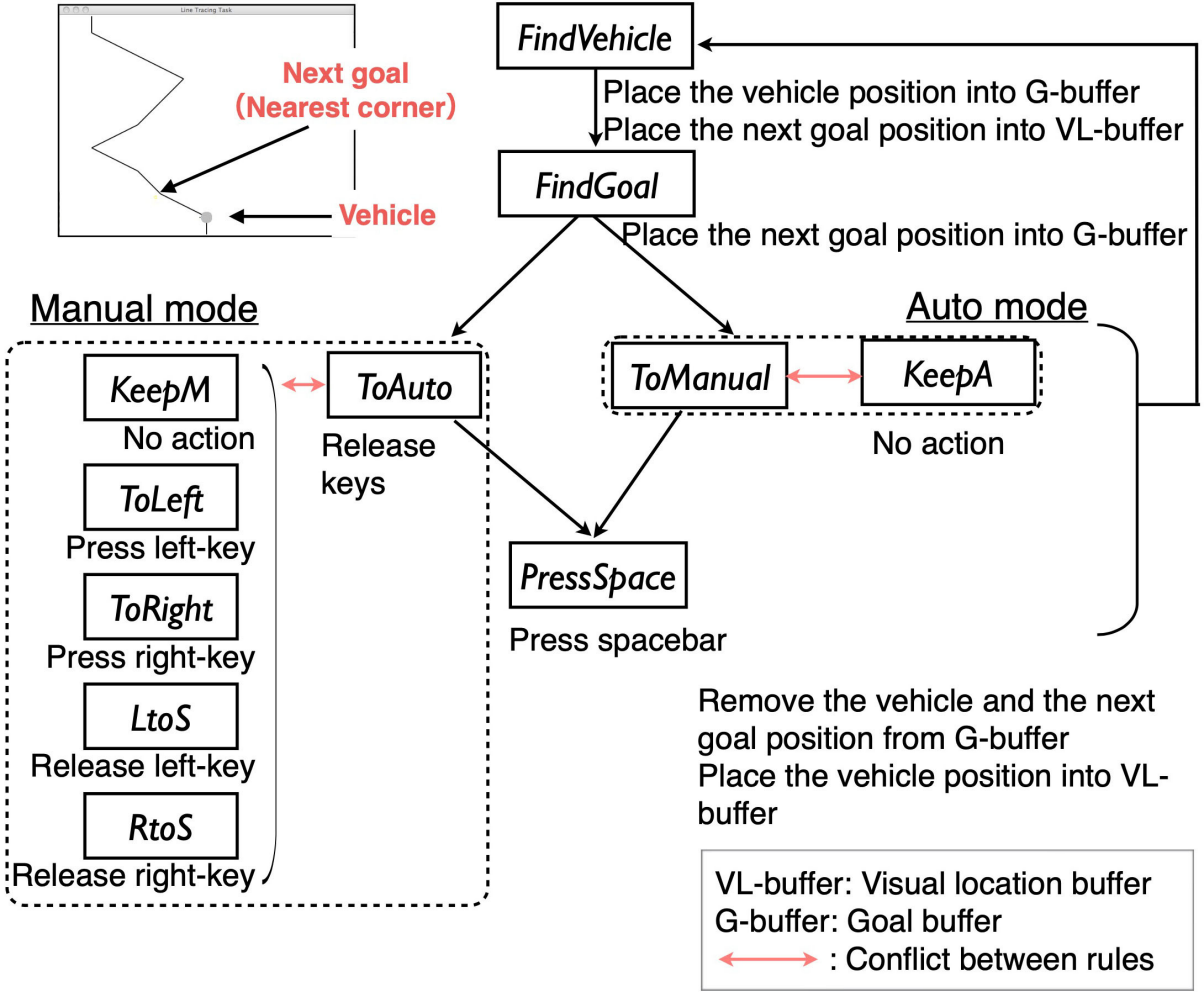
The basic cycle of the model.

Figure 4 describes the basic cycle of the model. The model uses the production, goal, vision, and motor modules of ACT-R 6, and 11 production rules. These rules consist of a perceptual (the top part of the figure) and motor process (the bottom part of the figure) similar to previous driving models in ACT-R (Salvucci, 2006; Ritter et al., 2007; Salvucci and Taatgen, 2010).

In the perceptual process, the model picks visual information from a visual location buffer that holds location information of objects in the environment. The *FindVehicle* rule finds the horizontal position of the vehicle and places it into the goal buffer. The *FindGoal* rule finds the horizontal position of the nearest goal position and places it into the goal buffer. The position information in the goal buffer is used in the subsequent motor process. After the motor process, the information in the goal buffer is cleared to begin the next cycle.

The motor process depends on the current mode. In each mode, there is a rule to switch the current mode (*ToAuto*/*ToManual*). These mode-switching rules send a command to release currently pressed keys to the motor module. After finishing the key-release, the *PressSpace* rule sends a motor command pressing the space-bar, toggling the mode.

To fire the mode-switching rules, the model needs to solve conflict resolution (Equations 1 and 2) with other rules in each situation. In the auto mode, the *ToManual* rule conflicts with the *KeepA* rule that just clears the goal buffer. In the manual mode, the *ToAuto* rule competes with the *KeepM, ToLeft, ToRight, LtoS*, and *RtoS* rules. These five rules have different conditions specifying the vehicle and the goal positions, and current move-commands (left, right, straight). The action clauses of the *ToLeft, ToRight, LtoS, RtoS* rules send a command to hold or release a key to the motor module^5^. The *KeepM* rule does not have any action clauses relating to the motor module. This rule just clears the goal buffer.

**Figure 5:**
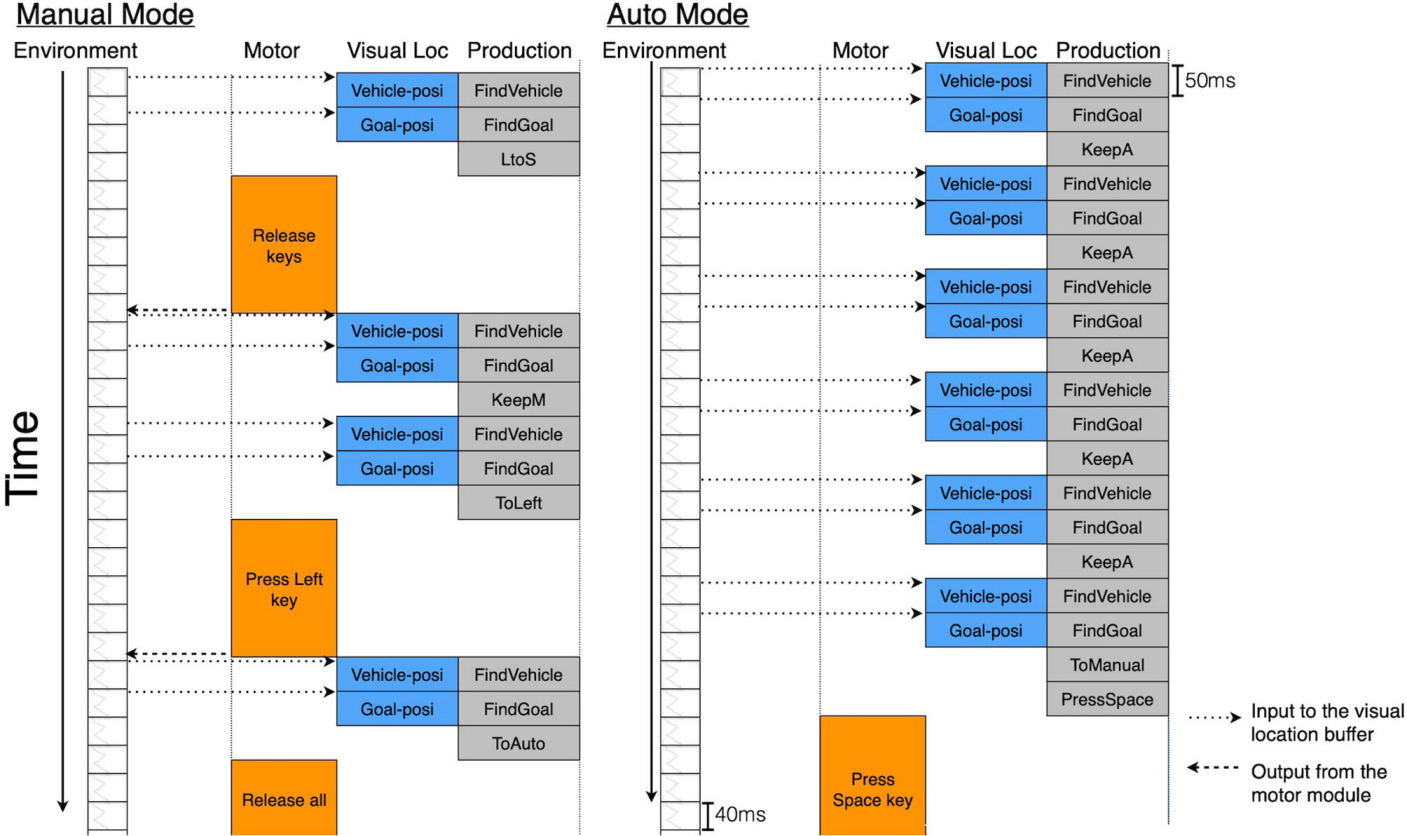
Time flow diagram of the ACT-R model components as they perform the task.

Figure 5 presents a time flow diagram showing the relations between the environmental changes and the model cycles. Each column of the diagram represents events of the environment and the modules of the model. The environment regularly updates the screen every 40 ms. Individual rule firings take 50 ms, but the cycle of the model is not regulated. There are delays due to the interaction with the environment. The processing of the visual location module itself has no delay. The model recognizes the vehicle or the goal position on the screen at the same time as the corresponding rule fires. However, the ACT-R motor module needs preparation and execution time, which depends on the status of the motor module. These delays disadvantage manual control by the ACT-R model compared to the automatic control in the task simulation.

### 4.4. Learning and Mode Switching

We now explain how the model adapts to the two modes. First, we describe the default mechanism of ACT-R and then our modification of the mechanism.

#### 4.4.1. Default Learning of ACT-R

In studies that use ACT-R reinforcement learning (Lovett and Anderson, 1996; Anderson et al., 2004; Anderson, 2007), a reward parameter is assigned to the specific rules by a modeler. When a rule with a reward value fires, all rules that have fired since the last utility update receive the reward. Following this paradigm, this study assigns a reward parameter to the *FindVehicle* rule (see Figure 4). In every cycle of the model, this rule perceives the vehicle location. Thus, it is reasonable to assume that this rule triggers rewards by checking a relative position between the vehicle and the line to follow.

More specifically, the default framework of ACT-R divides the *FindVeheicle* rules into two variations: the *FindVeheicleOnline* rule that fires when the vehicle is on the line, and the *FindVeheicleOffline* rule that fires when the vehicle is off the line. Then, it sets the reward value triggered by the former to positive, and the reward value triggered by the latter to zero. As a result, when the vehicle is on the line, the utilities of the rules included in the current mode rises, and when the vehicle is off the line, those utilities go down. As the *FindVeheicleOffline* rule keeps firing, the utility of the rules for mode switching (*ToManual* in the auto mode/*ToAuto* in the manual mode) becomes higher than the competing rules (*KeepA* in the auto mode/*KeepM, ToLeft, ToRight, LtoS, RtoS* in the manual mode), and causes the model to switch to another mode.

However, there are the following two difficulties for simply applying this paradigm to our model:

1. The problem of rewarding.
  As shown in Figure 5, there are motor delays in the ACT-R architecture. Reflecting these delays, the rules in the manual mode fire slightly less often than the automatic mode rules over similar time periods when they are preferred. They thus receive less opportunity for updating rewards.
2. Absence of direct comparisons.
  During the cycle in Figure 4, the utilities of the two modes are not directly compared. The rules keeping the current mode (*KeepA*/*KeepM*) receive rewards corresponding to how well the vehicle moves in the current mode. On the other hand, rewards for the mode-switching rules are influenced by the vehicle movement in both of the two modes because the perception-action cycle of mode-switching bridges across the two modes (see Figure 5). Accordingly, mode switching is made only based on the utility for the current mode without considering how well the vehicle moved in the other mode. Adaptation to the proper mode may be possible even if this problem exists. However, it is reasonably assumed that humans can predict future rewards by remembering past experiences.

#### 4.4.2. A Solution to the Problem of Rewarding

The present study uses the reinforcement learning method from SMDP to solve the problem of rewarding. Unlike the MDP situation where rewards are regularly given, in the SMDP situation, it is necessary to appropriately distribute the rewards given irregularly to the rules. According to Sutton and Barto (1998), the expectation value of the reward received at time *t* under SMDP is defined by Equation (3).

(3) $$\begin{array}{r} R\left(t\right)=\overset{T}{\underset{k=0}{\sum }}\gamma^{k}r_{t+k+1} \end{array}$$

*T* is the elapsed time since the last reward; γ is the time discount rate; *r* is the immediate reward received *k* units time ago. Because *T* is not fixed in the SMDP theory, the frequency of receiving rewards is possibly different between rules. To solve this, the above equation sums immediate rewards to balance the amount of rewards received over the long term. Following this definition, the present study assumes the travel distance of the vehicle per unit time (speed) as an immediate reward, and the model received the summation of the immediate reward across the interval of reward acquisition.

Specifically, distinguishing the *FindVehicleOnlineSMDP* rule that fires when the vehicle is on the line from the *FindVehicleOfflineSMDP* rule that fires when the vehicle is off the line, the reward is calculated as follows.

- The reward calculation of *FindVehicleOnlineSMDP*
  (4) $$\begin{array}{r} R_{t}=MaxVehicleSpeed\times DurationFromReward \end{array}$$
- The reward calculation of *FindVehicleOfflineSMDP*
  (5) $$\begin{array}{r} R_{t}=abs\left(vposi_{t}-vposi_{t-1}\right) \end{array}$$

In Equation (4), the maximum vehicle speed (*MaxVehicleSpeed*) is set to 48 pixels/s. The duration from the last reward (*DurationFromReward*) is estimated by using the temporal module of ACT-R, which is used to simulate the estimation of subjective time (Taatgen et al., 2004). In Equation (5), the vehicle position at the last reward (*vposi*_*t*−1_) is stored in the goal buffer and compared to the current vehicle position (*vposi*_*t*_).

Thus, these equations represent approximations of the travel distance of the vehicle from the previous reward to the current reward. The model cannot grasp the exact travel distance of the vehicle, because the vehicle position is recognized only when the *FindVehicle* rule fires.

#### 4.4.3. A Solution to the Problem of Conflict Resolution

To date, several researchers have investigated applications of reinforcement learning to complex tasks that are broken into several subtasks (Singh, 1992; Sutton et al., 1999; Doya et al., 2002). Gating is a mechanism used for such tasks. At each point of the task, the agent selects a subtask, and switches to use a corresponding module that independently learns the utility of the primitive action.

Subtasks in the present study correspond to auto and manual operation. To select these modes, a gating mechanism is applied before selecting the primitive rule. As represented in the EDFT model by Gao and Lee (2006), adaptation to an automated system is regarded as a problem of balancing between self-confidence (*SC*) of manual operation and trust (*T*) of an automation system. Therefore, in this study, we assumed that the gating mechanism compares *SC* and *T* to select the auto and the manual modes. In this research, these utility values are represented as competition between two rules that can be kept in ACT-R's production module. In our model, *SC* can be interpreted as a utility of a rule representing the manual mode. On the other hand, *T* can be regarded as a utility of a rule representing the auto mode. Thus, *KeepM* and *KeepA* correspond to *SC* and *T*, respectively. In this paper, we call this mechanism, what might be seen as a type of buffer holding a specific type of information, the “gate module”.

The gate module controls the gate that transitions the subtask. In our model, the transition between modes becomes possible only when the gate is opened. This mechanism can be implemented by including the following conditions in the *ToAuto* and *ToManual* rules.

- The condition of the gate module in *ToAuto*: *SC* < = *T*
- The condition of the gate module in *ToManual*: *SC* >= *T*

In other words, prior to comparing utilities of primitive rules, *SC* held by the gate module is compared with *T*. These values change only when the corresponding mode is selected. The value of *SC* decreases when the manual mode is selected and the vehicle is off the line. When the auto mode is selected and the vehicle is off the line, the value of *T* decreases. When the order relation between *SC* and *T* changes, the gate opens, and it becomes possible to fire the rule to switch the mode. By providing such a mechanism, the model is able to make decisions based not only on the utility within the current mode, but also with the mode used in the past.

## 5. Model Simulations

We now present two simulations of the model, demonstrating its performance on the two conditions in Maeghigashi et al. (2013) after presenting a test of our implementation of the line-following task.

### 5.1. Simulation 1: Base-Level Simulation

Before presenting a simulation involving the two modes, we conducted a simulation of Experiment 1 by Maeghigashi et al. (2013) to confirm the correspondence of the base performance of the auto and manual modes in the two implementations.

#### 5.1.1. Method

Maeghigashi et al. (2013) compared the performance of the manual mode with the auto mode for each corresponding *Ca* and *Cm* level. In their experiment 1, the participants conducted the task without using the auto control mode (Data-Manual: *n* = 65). We ran the model more than the number of participants because we wanted firm predictions from the model (Ritter et al., 2011).

Similarly, we ran the model with the initial control mode set to manual and removed the *ToAuto* rule from the model (Model-Manual: *n* = 1,000). We also compared baseline auto performance between the original environment implemented in Java (Java-Auto: *n* = 65) and the simulated environment implemented in ACT-R's interface, AGI (CL-Auto: *n* = 1,000). This comparison was performed to make sure our replicated task environment followed Maeghigashi et al. (2013)'s original environment.

#### 5.1.2. Results

**Figure 6:**
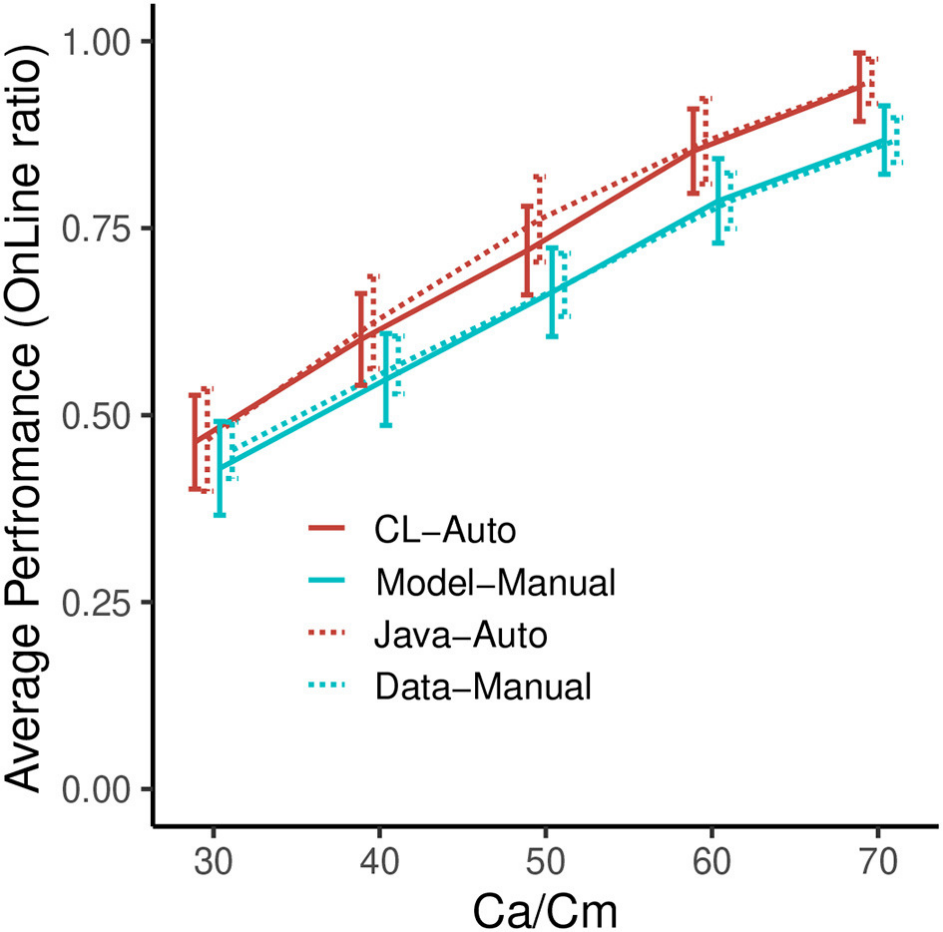
Performance of the model and the data in the baseline simulation. Error bars represent σ/2.

Figure 6 indicates the performances of the four conditions in each *Ca* and *Cm* level, showing the ratio of time that the vehicle is on the line. From this figure, it can be observed that the performance of all four lines increases with higher *Ca*/*Cm* levels, consistent with the manipulations of capability. In addition, we can confirm that the auto controls are better than the manual controls in both the experimental data and the simulation. This result illustrates the manual disadvantages in this task. Although the performance of the model is relatively lower than that of the data, the correlations between the experiment and the simulation are high (Auto: *r*^2^ = 0.993, *RMSE* = 0.022, Manual: *r*^2^ = 0.999, *RMSE* = 0.014)^6^. These results indicate that we succeeded in implementing a firm base for the simulation involving the choice of the two modes.

### 5.2. Simulation 2: Simulation With the Two Modes

This simulation is conducted to simulate Experiment 2 by Maeghigashi et al. (2013) that specifies the automation use ratio.

#### 5.2.1. Method

In Experiment 2, participants could use the auto control mode. The results were shown in Figure 3. In this simulation, to examine the adaptation process in this task, we manipulated the methods of rewarding and conflict resolution as follows.

- Rewarding
  – Default: The model receives a reward according to the method in section 4.4.1.
  – SMDP: The model receives a reward according to the method in section 4.4.2.
- Conflict resolution
  – Default: The model switches mode according to the method in section 4.3.
  – Gating: The model switches mode according to the method in section 4.4.3.

Combining the methods of rewarding and conflict resolution, four models [Default-Default, Gating-Default, Default-SMDP, Gating-SMDP] were prepared. Each model was run 500 times under the same conditions as the participants. All models have the default learning rate (*alpha* = 0.2), the default noise level (*egs* = 0)^7^, and the same initial utilities of rules (*utility*_*all*_ = 5). In the default rewarding models, the reward of the *FindVeheicleOnline* rule was set to 10, and the reward of the *FindVeheicleOffline* rule was set to 0.

#### 5.2.2. Results

**Figure 7:**
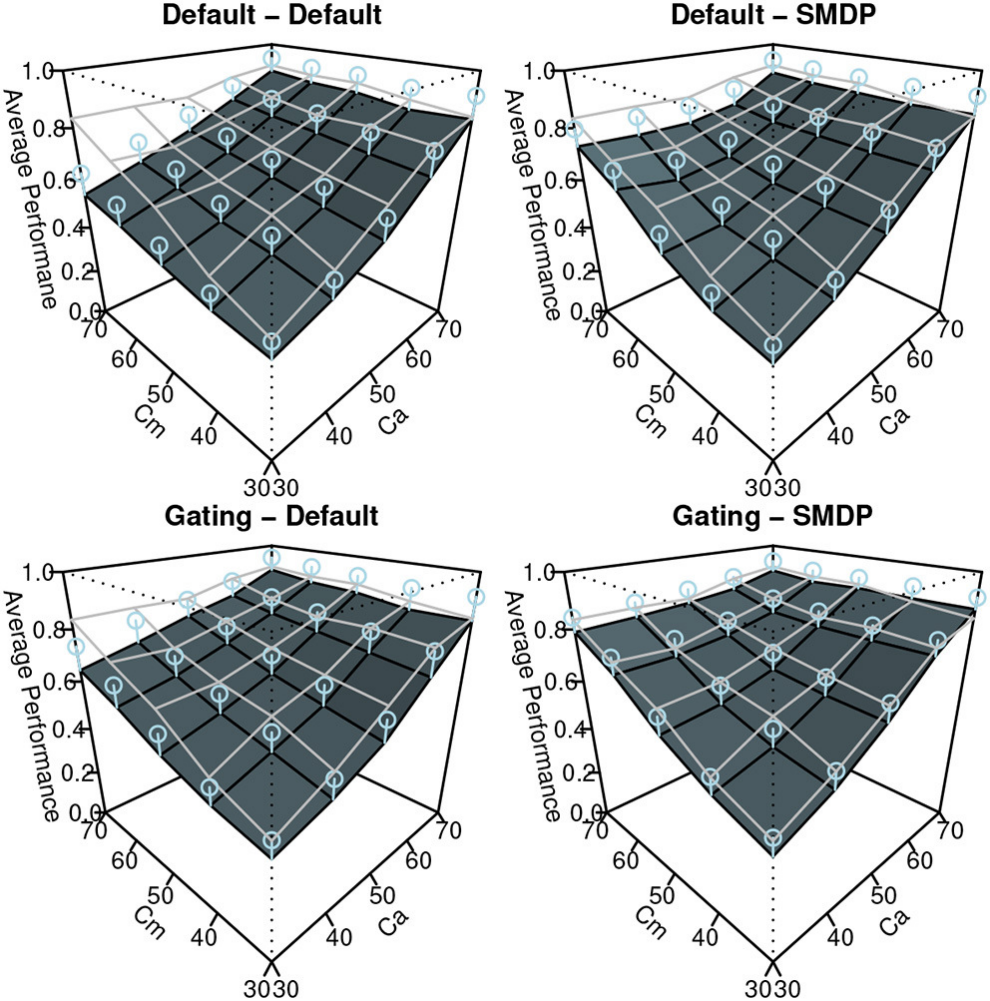
The results of the simulation (Performance). Error bars represent σ/2.

**Figure 8:**
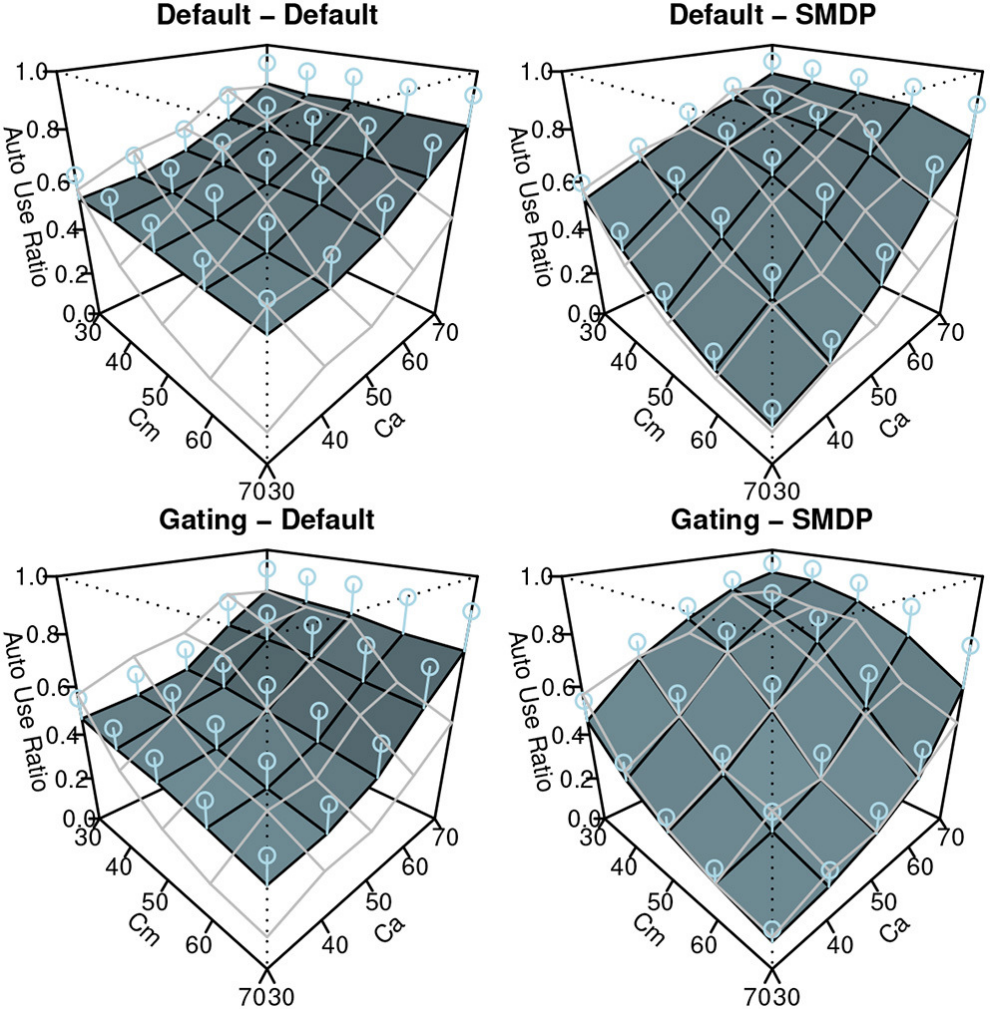
The results of the simulation (Auto Use Ratio). Error bars represent σ/2.

**Figure 9:**
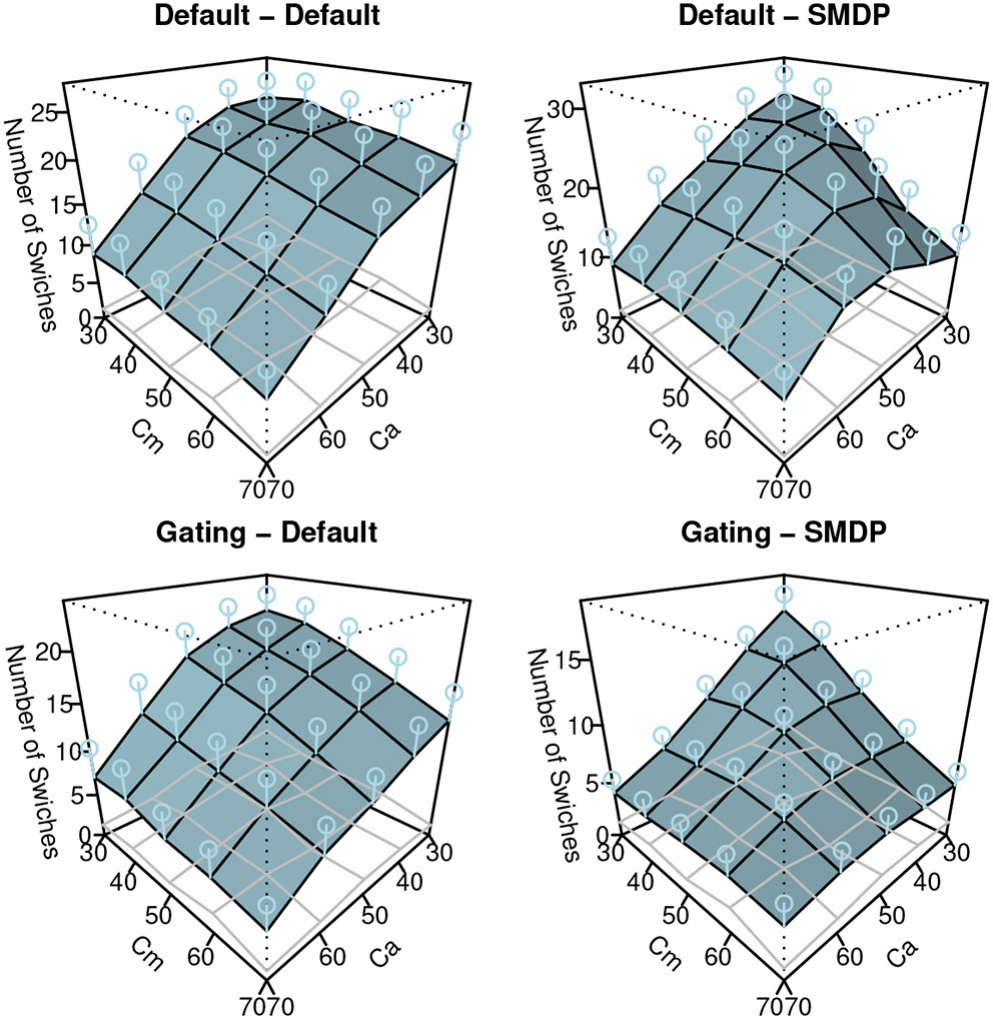
The results of the simulation (the number of switches). Error bars represent σ/2.

The results of the simulation are shown in Figures 7–9. Similar to Figure 3, these show the performance (the ratio of time that the vehicle was able to follow the line), the auto use ratio, and the number of mode switches, respectively. All figures contain the results of the four models with the green (dark) planes while the human participant data initially presented in Figure 3 are indicated with the meshed plane (the mesh being close to the planes indicates a good fit). Most of the models in the figures replicated the trends observed in the behavioral data: the increase of performance as *Ca* and *Cm* increase, the increase of the auto use ratio with as the *Cm* level decrease, the decrease of the auto use ratio as the *Ca* level increases, and the decrease of the number of switches as the *Ca* and *Cm* levels increase. However, the models without the SMDP rewarding failed to replicate the influence of the *Cm* level in the auto use ratio; these models were not influenced by the capability of the manual mode.

To quantify the fit between the model and the data, we computed *R*^2^ and the Root-Mean Square Errors (*RMSE*) as shown in Table 1. Although the degree of the fit varies between the three behavioral indices, we can observe the best fit of the model includes the two new model features (Gating-SMDP) for all the behavioral indices. This result generally supports the validity of our model. Especially in the performance and the automation use, the model with the two solutions achieved a high fit to the data. However as shown in the number of switches (Figure 9), there are still differences between the model and the participants; the model made more switches compared to the participants.

**Table 1:** Fit of the model predictions (*N* = 500) to the participant data (*N* = 63).

**Measurement**	**Models**	***R*^2^**	**RMSE**
Performance	Default-default	0.792	0.135
	Default-SMDP	0.926	0.107
	Gating-default	0.907	0.101
	Gating-SMDP	**0.969**	**0.053**
Auto use ratio	Default-default	0.462	0.213
	Default-SMDP	0.794	0.138
	Gating-default	0.618	0.149
	Gating-SMDP	**0.945**	**0.072**
Number of switches	Default-default	0.433	14.781
	Default-SMDP	0.683	15.585
	Gating-default	0.612	11.117
	Gating-SMDP	**0.798**	**6.004**

*The bold numbers indicate the best fit*.

## 6. Conclusion

The present study simulated adaptations of automated vehicle operation in a simple tracking task. We assumed that a general paradigm of reinforcement learning can be applied to this problem. Based on this assumption, we used the ACT-R architecture to explore mechanisms to simulate the human participant behavior data. The results of the simulation show overall correspondence with the experimental data, suggesting the validity or at least usefulness of our assumptions. In this final section, we presents implications and limitations of the study.

### 6.1. Implications

**Figure 10:**
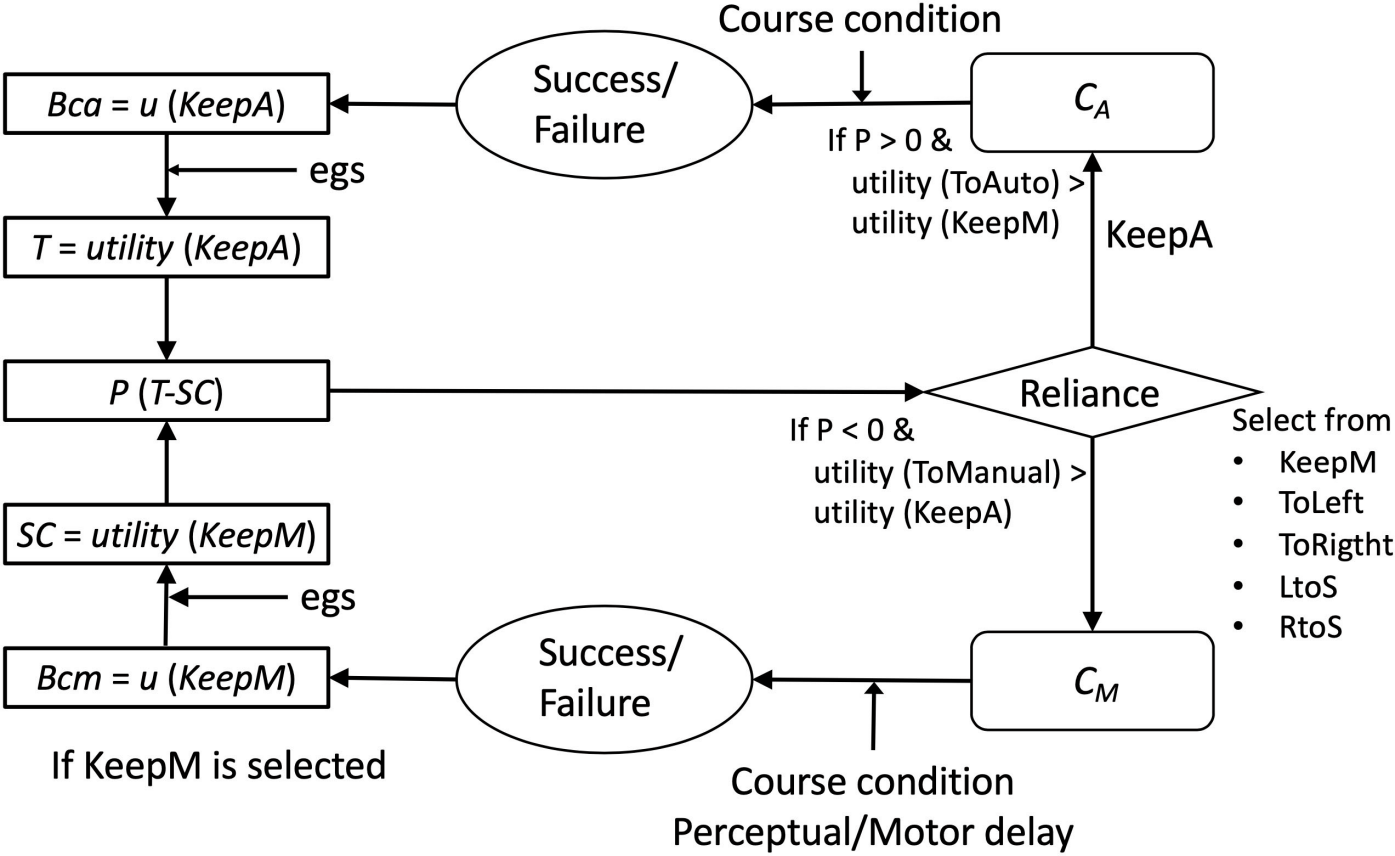
Correspondence with the EDFT model.

As an implication from the current study, we first claim the advantage of connecting an abstract theory (EDFT) to a cognitive process model (ACT-R). Figure 10 summarizes the process of our model (Gating-SMDP) in the framework of the EDFT theory. The utility module of ACT-R corresponds to Belief and Trust in the EDFT theory. However, unlike the previous model, our model has knowledge to execute the task and performs the task. Our model also does not perceive *Ca*/*Cm* directly. Randomized course conditions influence the performance (success/failure) of the task. Moreover, complex perceptual/motor factors are involved in manual mode performance. Therefore, the model's reliance on the automation interacts with the performance of the task in our model, which in turn influences reliance on the automation. As Bainbridge (1983) noted, to understand decision making about the use of automation, one needs to consider monitoring the performance of the auto and manual performance. This work is a first step to include performance factors into modeling the use of automation though there are still many limitations to applying it to actual systems in the real world.

The present work can also be seen as a contribution to both the field of reinforcement learning and ACT-R cognitive modeling. ACT-R has so far been used to simulate many psychological experiments. Because of this background, this architecture made it possible to produce a simulation that can be directly compared with the participant data. In our study, the perceptual and motor modules of ACT-R were used to represent the time constraints of the task (as shown in Figure 5). However, to simulate the human behavior, we needed to extend the reinforcement learning implemented in ACT-R. These extensions included the summation of rewards over time and the gating mechanism. Without these previously developed for SMDP (Sutton et al., 1999), we could not have achieved as good a fit to the data (Figures 7–9).

Combining these different modeling approaches allows us to predict human behavior in time-critical tasks. Despite the long history of this architecture, studies using reinforcement learning in ACT-R have not been so common. In this community, instance-based learning (IBL), which uses declarative knowledge as past problem-solving experiences, is more popular than reinforcement learning (Lebiere et al., 2007, 2009). However, because of the long time delay in retrieving declarative knowledge, IBL is not suited to model decision making in time-critical tasks, rather it is limited to modeling cognitive processes in discrete-time step tasks (MDP). On the other hand, reinforcement learning in SMDP has been mainly developed in the field of control engineering and robotics (e.g., Asada et al., 1999; Sutton et al., 1999; Doya et al., 2002; Elfwing et al., 2004), which was not directly aimed to make predictions of human behavior.

Contrary to the above previous research, our gating module enables a fast decision making process with a human-comparable time constraint represented in ACT-R. In addition, we hypothesize that this mechanism relates to topics of prediction in the sense of the free energy principle (Friston, 2010), which is a general brain theory integrating information theory, Bayesian inference, and reinforcement learning. In this framework, reinforcement learning in complex tasks is modeled with belief-based schemes (Friston et al., 2016), which calculates a future prediction based on a belief of the world constructed by past experience. Although our current model does not explicitly have a prediction process, the gating module plays a role to hold a belief of the world constructed from past experience. In other words, we consider that the gating module is necessary to include as a prediction process in reinforcement learning in ACT-R.

We also assume that by implementing the prediction process, we can extend our model to include more subtle emotional processes such as disappointment (Joffily and Coricelli, 2013) caused by a large prediction error. Such an emotional process based on a prediction possibly could improve the fit of our model especially in the number of switches.

In sum, our study has not only the practical merit of presenting modeling techniques for adaptations on automated vehicle operation but also the theoretical merit emerged from combining ACT-R with reinforcement learning theories.

### 6.2. Limitations and Future Study

We can also consider other several directions for future studies. The first direction is expanding the task to complex real-world situations. In this study, we chose an abstract simple task to be tractable in the default ACT-R architecture. However, to apply the findings to real-world situations, we need more realistic models of continuous control of steering and speed. To model such factors, we will need to add some functions converting the discrete action of production rules to continuous physical controls. We consider that such modeling can be accomplished by adding motor components in ACT-R like a previous model of driving (Salvucci, 2006).

It is also desirable to explore further factors influencing automation adaptation. In the real world, this problem is not purely a cognitive problem, but it includes social and ethical issues. Although it might not be easy to answer such a problem by cognitive modeling alone, we can accumulate understanding on the individual cognitive process to approach the problem. The other assumed factors influencing automation adaptation include mental workload (Rajaonah et al., 2008; Maehigashi et al., 2014), mental models (Beggiato and Krems, 2013), and the understandability of the system (Maehigashi et al., 2018). Such factors seem to be compatible with a cognitive architecture like ACT-R because it provides a way to represent mental models in declarative memory, which is utilized in IBL. The other possible factors involved in this problem such as anxiety or opportunism can also be modeled referring to recent studies of emotion representations in ACT-R (Dancy et al., 2015; Juvina et al., 2018; Van Vugt et al., 2018). We consider that combining such various levels of mental functions possibly provides a novel explanation of the complex nature of reliance on automation.

## Data Availability Statement

The raw data and model supporting the conclusions of this article will be made available by the authors, without undue reservation. The demo movie and the source code of the model are available at https://youtu.be/Kf3vIuZ2-QQ and https://github.com/j-morita-shizuoka/line-following-tak/, respectively.

## Author Contributions

JM constructed the model and summarized the paper. KM led the project. AM and HT corrected the behavioral data. KK implemented the experimental environment. FR corrected the model and the papers. All authors contributed to the article and approved the submitted version.

## Conflict of Interest

The authors declare that the research was conducted in the absence of any commercial or financial relationships that could be construed as a potential conflict of interest.

## Footnotes

^1^Therefore, the command rate is not influenced by a key-repeat rate setting in an operating system or keyboard.

^2^The original experiment (Maeghigashi et al., 2013), experiment 1, employed 27 participants, but we included additional data in the their later study (Maehigashi et al., 2014), experiment 1.

^3^This approach was originally developed by Card et al. (1983) based on the finding by Sternberg (1969). The perceptual-motor system of ACT-R was also developed based on EPIC architecture (Kieras and Meyer, 1997).

^4^The latest version of ACT-R is 7.14. We used version 6 because we first implemented the model in that version. The latest version has a richer interface but the basic structures and functions have not largely changed.

^5^The default implementation of ACT-R 6 does not include key-press and key-release functions (but see the extras folder for a new draft implementation of this capability). We used a customized module in which the time parameter of key-punch is used for these actions.

^6^Although we used the same algorithm and parameters as the original experiment, slight differences occurred in the automatic modes used in the two task implementations due to random factors such as the random number seed and the actual course segments chosen.

^7^In the ACT-R manual (Bothell, 2007), this parameter is *expected gain s*, the noise (s in Equation 1 and egs in the ACT-R system) added to the expected utility value of each rule.
